# Collective atomic scattering and motional effects in a dense coherent medium

**DOI:** 10.1038/ncomms11039

**Published:** 2016-03-17

**Authors:** S. L. Bromley, B. Zhu, M. Bishof, X. Zhang, T. Bothwell, J. Schachenmayer, T. L. Nicholson, R. Kaiser, S. F. Yelin, M. D. Lukin, A. M. Rey, J. Ye

**Affiliations:** 1 JILA, NIST and Department of Physics, University of Colorado, 440 UCB, Boulder, Colorado 80309, USA; 2 Université de Nice Sophia Antipolis, CNRS, Institut Non-Linéaire de Nice, UMR 7335, F-06560 Valbonne, France; 3 Department of Physics, University of Connecticut, Storrs, Connecticut 06269, USA; 4 Department of Physics, Harvard University, Cambridge, Massachussetts 02138, USA

## Abstract

We investigate collective emission from coherently driven ultracold ^88^Sr atoms. We perform two sets of experiments using a strong and weak transition that are insensitive and sensitive, respectively, to atomic motion at 1 μK. We observe highly directional forward emission with a peak intensity that is enhanced, for the strong transition, by **>**10^3^ compared with that in the transverse direction. This is accompanied by substantial broadening of spectral lines. For the weak transition, the forward enhancement is substantially reduced due to motion. Meanwhile, a density-dependent frequency shift of the weak transition (∼10% of the natural linewidth) is observed. In contrast, this shift is suppressed to **<**1% of the natural linewidth for the strong transition. Along the transverse direction, we observe strong polarization dependences of the fluorescence intensity and line broadening for both transitions. The measurements are reproduced with a theoretical model treating the atoms as coherent, interacting radiating dipoles.

## Abstract (web summary)

Light scattering from a dense coherent medium is determined by the interplay of dispersive and radiative dipole–dipole interactions. Here, the authors control the motional effects that obscure the coherence of scattered light and study collective emission in a driven gas of cold strontium-88 atoms.

## Graphical abstract

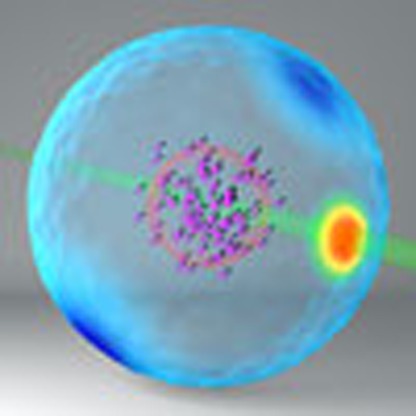

Understanding interactions between light and matter in a dense atomic medium is a long-standing problem in physical science^1^,^2^ since the seminal work of Dicke^3^. In addition to their fundamental importance in optical physics, such interactions play a central role in enabling a range of new quantum technologies including optical lattice atomic clocks^4^ and quantum networks^5^.

The key ingredient in a dense sample is dipole–dipole interactions that arise from the exchange of virtual photons with dispersive and radiative contributions, and their relative magnitude varies between the near-field and far-field regimes. The dispersive (real) part is responsible for collective level shifts and the radiative (imaginary) part gives rise to line broadening and collective superradiant emission^6^,^7^,^8^. Intense theoretical efforts have been undertaken over many years, to treat the complex interplay between the dispersive and radiative dynamics^9^,^10^,^11^,^12^,^13^,^14^,^15^,^16^,^17^,^18^. However, experimental demonstrations that provide a complete picture to clarify these interactions have been elusive.

Collective level shifts and line broadening arising from the real and imaginary parts of dipole–dipole interactions have recently been observed in both atomic^19^,^20^,^21^,^22^,^23^ and condensed matter^24^ systems. The modification of radiative decay dynamics at low excitation levels has also been observed using short probe pulses^25^,^26^,^27^,^28^, and interaction effects were manifested in coherent backscattering^29^,^30^. Although simple models of incoherent radiation transport have often been used to describe light propagation through opaque media^31^,^32^ and radiation trapping in laser cooling of dense atomic samples^33^, coherent effects arising from atom–atom interactions, which are necessary to capture correlated many-body quantum behaviour induced by dipolar exchange, are beginning to play a central role. For example, the dipole–dipole interaction is responsible for the observed dipolar blockade and collective excitations in Rydberg atoms^34^,^35^,^36^,^37^,^38^,^39^,^40^,^41^; it may also place a limit to the accuracy of an optical lattice clock and will require non-trivial lattice geometries to overcome the resulting frequency shift^42^. Previous theoretical efforts have already shown that physical conditions such as finite sample size, sample geometry and the simultaneous presence of dispersive and radiative parts can play crucial roles in atomic emission^10^,^11^,^12^,^13^,^43^,^44^,^45^.

In this work we use millions of Sr atoms in optically thick ensembles, taking advantage of the unique level structure of Sr to address motional effects, to study these radiative and dispersive parts simultaneously. We demonstrate that a single, self-consistent, microscopic theory model can provide a unifying picture for the majority of our observations. These understandings can help underpin emerging applications based on many-body quantum science, such as lattice-based optical atomic clocks^4^,^46^,^47^, quantum nonlinear optics^39^, quantum simulations^48^ and atomic ensemble-based quantum memories^49^.

## Results

### Experimental setup

**Figure 1:**
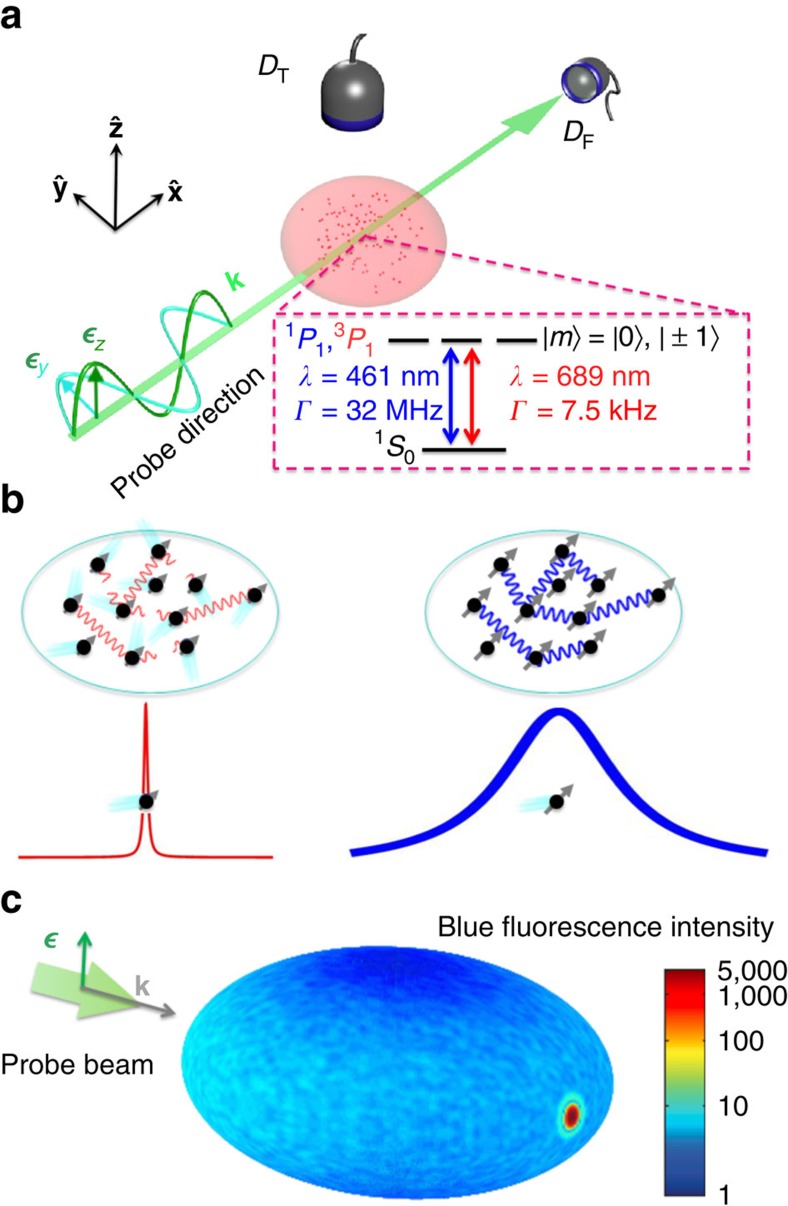
The experimental scheme and concept. (**a**) We weakly excite the strontium atoms with a linearly polarized probe beam and measure the fluorescence with two detectors: one in the forward direction, 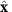, and the other almost in the perpendicular direction, 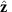. We probe two different *J*=0 to *J*'=1 transitions. The first transition is a ^1^*S*_0_−^1^*P*_1_ blue transition with a natural linewidth of *Γ*=32 MHz and the second is a ^1^*S*_0_−^3^*P*_1_ red transition with *Γ*=7.5 kHz. (**b**) In the coherent dipole model, photons are shared between atoms. When the Doppler broadened linewidth becomes comparable to the natural linewidth, dephasing must be considered. At our ∼1 μK temperatures the Doppler broadening is ≈40 kHz, meaning motional effects are important only for the red transition. (**c**) The three-dimensional intensity distribution predicted for a blue probe beam. The coupled dipole model predicts a strong 10^3^ enhancement of the forward intensity compared with other directions and a finite fluorescence along a direction parallel to the incident polarization. The speckled pattern is due to randomly positioned atoms and can be removed by averaging over multiple atom configurations.

Bosonic alkaline-earth atoms with zero nuclear spin have simple atomic structure compared with the more complex hyperfine structure present in typical alkali metal atoms that complicates the modelling and interpretation of the experimental observations. For example, ^88^Sr atoms have both a strong ^1^*S*_0_−^1^*P*_1_ blue transition (*λ*=461 nm) and a spin-forbidden weak ^1^*S*_0_−^3^*P*_1_ red transition (*λ*=689 nm), with a strict four-level geometry (Fig. 1a). When the atoms are cooled to a temperature of ∼1 μK, Doppler broadening at 461 nm is ∼55 kHz, which is almost three orders of magnitude smaller than the blue transition natural linewidth, *Γ*=32 MHz. To an excellent approximation, atomic motion is negligible for atomic coherence prepared by the 461-nm light. To the contrary, the red transition with a natural linewidth *Γ*=7.5 kHz is strongly affected by atomic motion. By comparing the behaviours of the same atomic ensemble observed at these two different wavelengths (Fig. 1b), we can thus collect clear signatures of motional effects on coherent scattering and dipolar coupling^50^,^51^.

We use the experimental scheme shown in Fig. 1a, to perform a comprehensive set of measurements of fluorescence intensity emitted by a dense sample of ^88^Sr atoms. The sample is released from the trap and then illuminated with a weak probe laser. We vary the atomic density, cloud geometry, observation direction and polarization state of the laser field, and we report the system characteristics using three key parameters as follows: the peak scattered intensity, the linewidth broadening and the line centre shift. For example, along the forward and transverse directions we observe different values of intensity and linewidth broadening, as well as their dependence on light polarization (see Fig. 1c). We also observe motional effects on the red transition in contrast to the same measurements on the blue transition.

**Figure 2:**
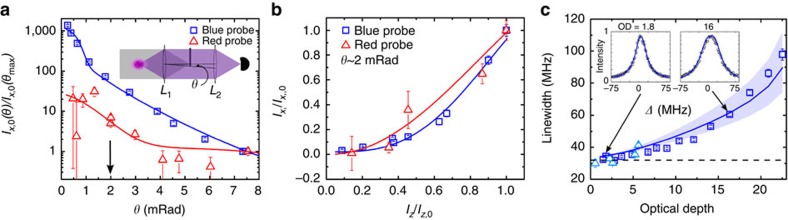
Forward scattering. (**a**) Comparison of forward scattering intensity versus angle using a red and blue probe beam. We use the setup shown in the inset, to block the probe beam. After interacting with the atoms the probe beam is focused using a lens, which also collimates the fluorescence from the atoms. We block the probe beam using a beam stopper, which we translate perpendicular to the probe beam, to change the angular range of fluorescence collected by the detector, characterized by the angle (*θ*) between 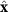 and the edge of the beam stopper (see Methods). The measured intensity, *I*_*x*,0_(*θ*), for each probe beam is normalized to the intensity at *θ*_max_=7.5 mRad. The dephasing caused by motion reduces the forward intensity peak for the red transition. (**b**) Comparison of intensity in the forward direction, *I*_*x*_, versus intensity in the transverse direction, *I*_*z*_. Both are varied by changing *N*. All measurements are made at *θ*=2 mRad (arrow in **a**) and normalized to the intensity, *I*_*x*,0_, for the atom number used in **a**. (**c**) Linewidth broadening in the forward direction measured by scanning the blue probe beam detuning, *Δ*, across resonance. Example lineshapes for different ODs are shown in the inset. Two different atom numbers are used, *N*=1.7(2) × 10^7^ (blue squares) and *N*/4 (cyan triangles). The dashed line represents *Γ* for reference. All solid curves are based on the full theory of coupled dipoles and the band in **c** is for a ±20% atom number uncertainty. All error bars are for statistical uncertainties.

In the experiment, up to 20 million ^88^Sr atoms are cooled to ∼1 μK in a two-stage magneto-optical trap, the first based on the blue transition and the second on the red transition. The atomic cloud is then released from the magneto-optical trap and allowed to expand for a variable time of flight (TOF), which allows us to control its optical depth and density. They are subsequently illuminated for 50(100)  μs with a large-size probe beam resonant with the blue (red) transition (Fig. 2a). The resulting scattered light is measured with two detectors far away from the cloud (see Fig. 1a). One detector is along the forward direction 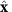 (detector *D*_F_) and the other along the transverse direction 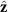 (detector *D*_T_, offset by ∼10°). For a short TOF, the atomic cloud is anisotropic and has an approximately Gaussian distribution with an aspect ratio of *R*_*x*_:*R*_*y*_:*R*_*z*_=2:2:1, where *R*_{*x*,*y*,*z*}_ are the root-mean-squared radii. We define OD as the on resonance optical depth of the cloud, 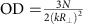, where *R*_⊥_ depends on the direction of observation with *R*_⊥,T_=*R*_*x*_=*R*_*y*_ and *R*_⊥,F_=(*R*_*z*_*R*_*y*_)^1/2^ for the transverse and forward directions respectively, *N* is the atom number and *k* is the laser wavevector for the atomic transition (see Supplementary Note 1).

### Forward observations

The coherent effect manifests itself most clearly in the forward direction (Fig. 2). To separate the forward fluorescence from the probe beam, we focus the probe with a lens (*L*_1_) after it has passed through the atomic cloud and then block it with a beam stopping blade, which can be translated perpendicular to the probe beam (Fig. 2a inset). The same lens (*L*_1_) also collimates the atomic fluorescence so that it can be imaged onto *D*_F_. The position of the beam stopper can be used to vary the angular range of collected fluorescence, characterized by the angle (*θ*) between 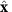 and the edge of the beam stopper (see Methods). Using the maximum atom number available in the experiment, the measured intensity *I*_*x*,0_(*θ*) is normalized to that collected at *θ*_max_=7.5 mRad. Both the blue (square) and red (triangle) transition results are displayed in Fig. 2a. For the blue transition, we observe a 1,000-fold enhancement of the normalized intensity for *θ*<0.5 mRad. The enhancement is also present for the red transition, but it is reduced by nearly two orders of magnitude at small *θ* due to the motional effect. On the other hand, the wider angular area of enhancement is attributed to the longer wavelength of the red transition. The forward intensity strongly depends on the atom number. In Fig. 2b, we present measurements of the forward intensity *I*_*x*_ versus the transverse intensity *I*_*z*_ at a fixed *θ*=2 mRad for different atom numbers. The intensities are normalized to those obtained at the peak atom number as used in Fig. 2a. To the first-order approximation, the transverse fluorescence intensity scales linearly with the atom number. Hence, the forward intensity of both the blue and red transitions scales approximately with the atom number squared.

In the forward direction, we have also investigated the linewidth broadening of the blue transition as a function of the atomic OD. By scanning the probe frequency across resonance, we extract the fluorescence linewidth, which is found to be determined primarily by the OD of the cloud (open squares in Fig. 2c). For the range of 0<OD<20, the lineshape is Lorentzian (see insets); however, the observed lineshape starts to flatten at the centre for OD>20. We have also varied the atom number by a factor of four, and to an excellent approximation the linewidth data are observed to collapse to the same curve when plotted as a function of OD (open triangles).

### Transverse observations

**Figure 3:**
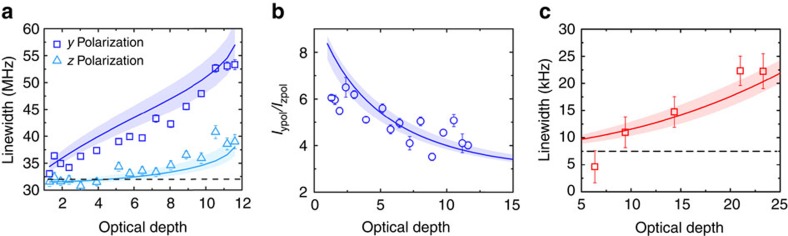
Transverse scattering. (**a**) Linewidth broadening for the blue transition in the transverse direction for 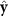 polarization (open squares) and 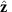 polarization (open triangles). (**b**) Intensity ratio, *I*_ypol_/*I*_zpol_, of 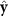 polarization to 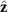 polarization measured in the transverse direction when a blue probe beam is used. For low optical depths single particle scattering is dominant and for single particle scattering almost zero intensity is predicted for 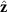-polarized fluorescence, as this polarization points directly into the detector. (**c**) Linewidth broadening for the red transition in the transverse direction for 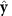-polarized light, showing a similar trend to the blue transition. This transition is more sensitive to magnetic fields; thus, a large magnetic field is applied to probe only the *m*=0 to *m*'=0 transition. All solid curves are based on the full theory of coupled dipoles and the band in **a**, **b** and **c** is for a ±20% atom number uncertainty. All error bars are for statistical uncertainties.

For independent emitters, the forward fluorescence should have no dependence on the probe beam polarization; however, the transverse fluorescence (along 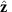) should be highly sensitive to the probe polarization and it is even classically forbidden if the probe is 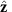 polarized. However, multiple scattering processes with dipolar interactions can completely modify this picture by redistributing the atomic population in the three excited magnetic states and thus scrambling the polarization of the emitted fluorescence. Consequently, even for a 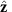-polarized probe there should be a finite emission along 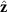 (see Fig. 1c), with an intensity that increases with increasing OD. Our experimental investigation of the fluorescence properties along the transverse direction is summarized in Fig. 3. Under the same OD along 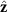, the 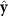-polarized probe beam (square) gives rise to a much more broadened lineshape for the blue transition than the 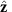-polarized probe beam does (triangle), as shown in Fig. 3a. Meanwhile, the peak intensity ratio of *I*_ypol_/I_zpol_ decreases significantly with an increasing OD, indicating the rapidly rising fluorescence with a 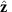-polarized probe when OD increases (Fig. 3b). For the red transition, the existence of Doppler broadening requires the lineshape data to be fitted to a Voigt profile. With the Doppler linewidth *Δ*_D_ fixed from the thermal velocity measured in free expansion, the Voigt profile determines the line centre and the Lorentzian linewidth with the Gaussian linewidth determined by the temperature. Figure 3c displays the Lorentzian linewidth obtained with a 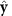-polarized red probe showing a strong increase of the linewidth with OD.

### Spectral broadening and shift

**Figure 4:**
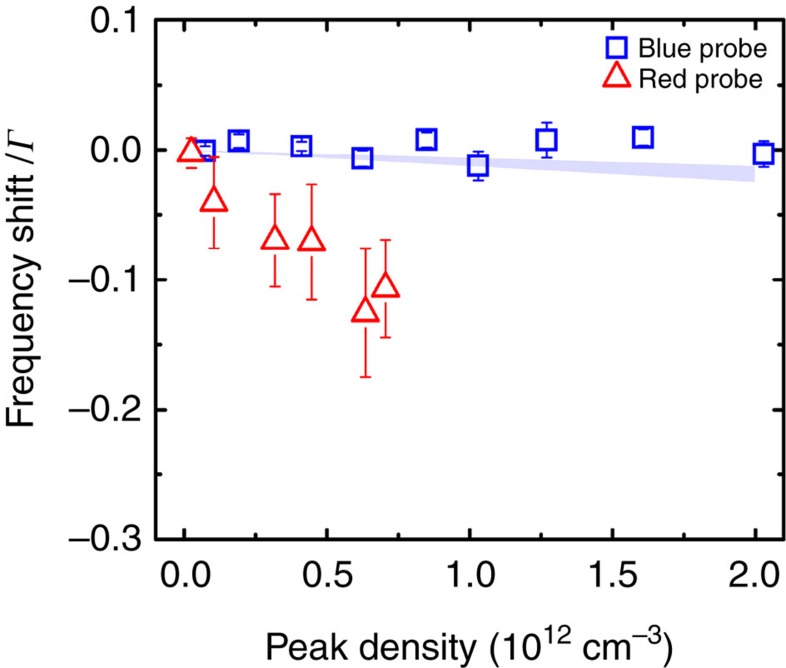
Frequency shift. Comparison of frequency shift normalized to the corresponding natural linewidth for the blue and red transitions. The blue frequency shift is consistent with 0–0.004 of *Γ* at an atomic density of 10^12^ cm^−3^. The red shift, on the other hand, shows more than 0.1*Γ* at densities up to 0.7 × 10^12^ cm^−3^.

To a good approximation, the dependence of the linewidth on OD along the forward and transverse directions (for the classically allowed 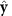 polarization in the single scattering limit) is similar. However, owing to the anisotropic aspect ratio of the cloud, for the same TOF, the OD is lower along 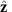 than along 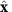. This is responsible for the smaller broadenings measured along 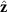 than along 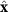. The classically forbidden polarization direction, on the other hand, exhibits a different scaling with OD, which is understandable given that the emission in this case comes only from multiple scattering events with dipolar interactions. The transverse linewidth broadening for the red transition is similar to that of the blue, and it does not depend sensitively on motional effects. This behaviour is in stark contrast to another important observation: the shift of the transition centre frequency. Figure 4 contrasts the linecentre frequency shift observed for ^1^*S*_0_−^1^*P*_1_ (square) and ^1^*S*_0_−^3^*P*_1_ (triangle, with original data reported in ref. 52 and see Supplementary Fig. 1). The blue transition frequency shift is consistent with zero at the level of 0.004*Γ* using an atomic density of 10^12^ cm^−3^. However, the measured density shift for the red transition (normalized to the transition linewidth) is more than one order of magnitude larger. This density-related frequency shift significantly exceeds the predicted value based on general *S*-matrix calculations of *s*-wave collisions^52^ (2.18 × 10^−10^ Hz cm^3^ if the unitary limit is used).

### Theory model

Before we turn to a microscopic model to obtain a full and consistent understanding of all these related experimental observations, we note that semiclassical models^53^ treating the atomic cloud as a continuous medium of an appropriate refractive index can give an intuitive explanation of the linewidth broadening in the forward direction. Classically, an incoming electric field is attenuated as it propagates through the medium according to the Beer–Lambert law and the forward fluorescence intensity is determined by the same mechanism. This simple semiclassical model recovers the linear dependence of the forward width for small OD and predicts a nonlinear dependence of the linewidth for large OD and a flattening of the line centre. However, we find that this semiclassical approach cannot provide explanations for most aspects of the experimental observations.

The full microscopic model builds on a set of coherently coupled dipoles. Here, each four-level atom is treated as a discrete radiating dipole located at a frozen position, but coupled with retarded dipole radiation, and it is driven with a weak incident laser beam. The atomic ensemble follows the Gaussian distribution observed in the experiment with the appropriate aspect ratio. By solving the master equation in the steady state, we find that the coherence, 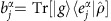, of atom *j*, located at **r**_*j*_ is modified by other atoms as^18^,^54^,^55^,^56^,^57^,^58^,^59^,^60^:


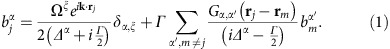


Here, 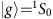, 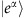 corresponds to the three excited states of ^1^*P*_1_ or ^3^*P*_1_, with *α*∈{*x*, *y*, *z*} representing the Cartesian states. In addition, 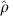 is the reduced density matrix of the atoms and *δ*_*γ*,*γ*′_ is the Kronecker Delta. The driving laser's linear polarization state *ξ* is along 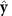 or 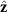, with wavevector **k** along 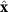, Rabi frequency Ω^*ξ*^ and detuned by *Δ*^*α*^ from the 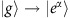 transition. The function *G*_*α*,*α*′_(**r**) accounts for the retarded pairwise dipolar interactions and is given by^18^,^48^,^56^ 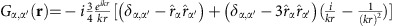. The fluorescence intensity 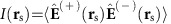, detected at position **r**_s_, can be determined^17^,^18^ as a function of 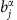,


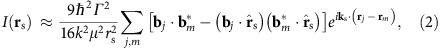


with *μ* is the atomic transition dipole moment and 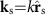.

## Discussion

To understand the forward enhancement we first consider non-interacting atoms under the zeroth order approximation. The atomic coherence is driven only by the probe field that imprints its phase and polarization onto the atoms: 
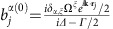
, where *Δ*=*Δ^α^*. The corresponding intensity, 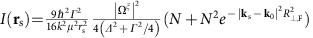 has a Lorentzian profile. It also exhibits an *N*^2^ scaling and an enhanced forward emission lobe, with an angular width given by the ratio between the transition wavelength and the transverse size of the sample 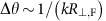. The forward lobe reflects the constructive interference of the coherently emitted radiation stimulated by the laser. Outside the coherent lobe the constructive interference is quickly reduced due to the random position of atoms^28^,^59^,^61^. The longer wavelength of the red transition corresponds to a wider angular width of the forward lobe for the red fluorescence.

Simple considerations can also give rise to a qualitative understanding of atomic motion-related effects on forward enhancement. Again for the red transition, the Doppler effect introduces random phases accumulated by 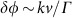. Here, *v* is the thermal velocity. The dephasing reduces coherent photon emission and gives rise to a net suppression of the forward emission intensity. The suppression becomes stronger with *Δ*_D_/*Γ*, with 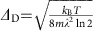 the Doppler width. Such a suppression is clearly observed for the red transition.

To address the linewidth broadening we now consider atoms coupled by dipolar interactions, which tend to emit collectively in an optically dense cloud. The collective emission manifests itself with a broader fluorescence linewidth. Moving to the first-order approximation, we note that the atomic coherence acquires contributions not only from the probe beam but also from the surrounding atoms, with 
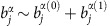
. Here, 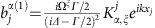 and 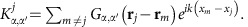 For a relatively dilute cloud with average interparticle distance 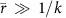, the far-field interactions dominate; thus, higher-order terms beyond 1/*r* can be neglected. Dipolar interactions modify the fluorescence lineshape, with consequences of both a frequency shift that depends on the cloud peak density *n*_0_ and a line broadening that is proportional to OD: 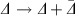 and 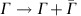, with 
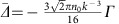
 and 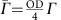. Thus, the first-order approximation provides an intuitive picture about the role of dipolar effects on the lineshape.

However, in a cloud with an increasingly large OD, dipolar interactions are stronger and multiple scattering processes become relevant. The first-order perturbative analysis then breaks down^62^,^63^,^64^. The full solution of equation (1) based on the coherent coupled dipole model becomes necessary to account for multiple scattering processes (see Methods). The first signatures arise from the forward fluorescence intensity, where its naive *N*^2^ scaling is reduced with an increasing atom number as a consequence of multiple scattering processes. This effect is observed in both red and blue calculations, and it is expected to be more pronounced on the red transition due to its longer wavelength. However, atomic motion leads to a lower effective OD, which tends to suppress multiple scattering processes and thus helps to partially recover the collective enhancement. The solid lines in Fig. 2a,b represent such quantitative theory calculations for both transitions, which agree with the experiment.

Meanwhile, for the linewidth broadening observed in the forward direction, it becomes evident that the scaling of the linewidth versus OD turns nonlinear at large values of OD. The experimental data falls within the shaded area in Fig. 2c, which represents the full solution with a 20% uncertainty in the experimental atom number. Multiple scattering processes are also key to the explanation of the measured fluorescence along the transverse direction, especially for the classically forbidden polarization 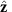. Indeed, for both intensity and linewidth broadening observed in the transverse direction, under either 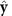 or 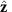 probe polarization, the full model (shown as shaded areas in both Fig. 3a,b) reproduces well the experimental results on ^1^*S*_0_−^1^*P*_1_. Taking into account motional dephasing (see Supplementary Note 2), the transverse broadening for ^1^*S*_0_−^3^*P*_1_ is also well reproduced as shown in Fig. 3c.

So far, we have shown the observed effects on linewidth and fluorescence intensity are uniquely determined by OD. However, following the arguments discussed above, the frequency shift arising from the dipolar coupling is expected to scale with atomic density, 
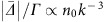
, which includes both the collective Lamb shift and the Lorentz–Lorenz shift^50^,^65^. For our experimental density, this effect is ≲10^−3^, which is consistent with the observed frequency shift for the blue transition (Fig. 4). (It is noteworthy that the role of multiple scattering processes is to further suppress this frequency shift mechanism^50^.) In contrast, for the red transition the measured density shift (normalized to *Γ*) is significantly larger than what is predicted from the current treatment of interacting dipoles; it is also much bigger than the unitarity limit of *s*-wave scattering. Qualitatively, we expect that as the atoms move and approach each other, the long-lived ground-excited state coherence in the red transition can be significantly modified by the collisional process and open higher partial wave channels. We can thus expect a larger collisional phase shift. This process can be further complicated by atomic recoil, light forces and Doppler dephasing^66^.

We have shown that a coherent dipole model describes light scattering in a dense atomic medium with collective effects and multiple scatterings. The model captures the quantitative features of the experimental observations. Motional effects, as manifested in dephasing, can be captured in the model as well. Our results provide useful guides for further developments of optical atomic clocks and other applications involving dense atomic ensembles.

## Methods

### Coherent dipole model

Here we present the derivation of equation (1). The fundamental assumption is to treat the atoms as frozen during the interrogation. This is an excellent approximation if *ħΓ* is much faster than other energy scales in the problem. The latter condition is always satisfied in the case of the blue transition. For the *J*=0 to *J*=1 configuration exhibited by ^88^Sr, we can label the *J*=0 ground state as 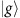 and the excited *J*=1 states using a Cartesian basis 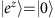, 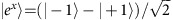, 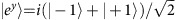. Here, the |0, ±1〉 states are the standard angular momentum ones. In the Cartesian basis, the vector transition operator for the *j* atom located at **r**_*j*_ can be written as 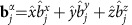. Here 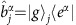. On this basis, the master equation governing the evolution of the reduced density matrix of the *N* atom ensemble, 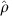, in the presence of an incident laser beam with linear polarization *ξ* can be written as^18^:


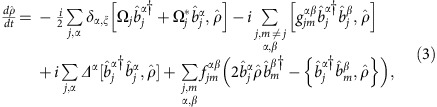


where 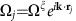 is the Rabi frequecy of the incident field, polarized along *ξ* 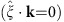 and detuned by *Δ*^*α*^ from the atomic transition 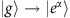. The parameters 
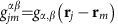
 and 
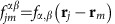
 are the components of the elastic and inelastic dipolar interactions between a pair of atoms at position **r**_*j*_ and **r**_*m*_, respectively, and are given by


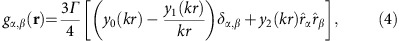



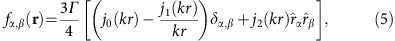


where 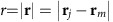, *y*_*n*_(*x*), *j*_*n*_(*x*) are the spherical Bessel functions of the second and first kind, respectively. Here, also *α*, *β*=*x*, *y* or *z* represent Cartesian components. The symbol *δ*_*γ*,*γ*′_ is the Kronecker Delta. In the low-intensity limit, we can project the density matrix into a state space including the ground state 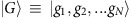 and states with only one excitation^57^,^58^,^59^ such as 
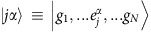
. In this reduced space, the relevant equations of motion simplify to


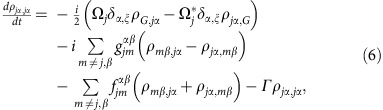



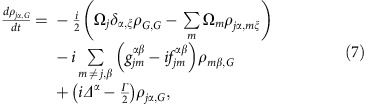



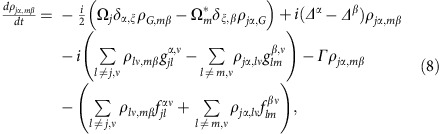



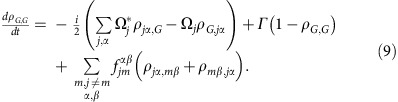


where 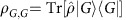, 
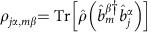
 and 
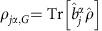
.

As we are interested in the situation of a weak probe limit, 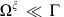, we expand the density matrix in successive orders of Ω^*ξ*^/*Γ*, 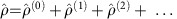, and keep the first-order terms. At this level of approximation, *ρ*_*G*,*G*_=1, *ρ*_*jα*,*mβ*_=0 and only the optical coherences 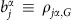 evolve in time accordingly to the following set of linear equations:


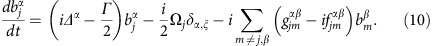


Here, *G*_*α*,*β*_(**r**)=(*f*_*α*,*β*_(**r**)+*ig*_*α*,*β*_(**r**))/*Γ*. The steady-state solution can be obtained by setting 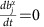 and then solving the subsequent 3*N* linear equations.

### Measure the enhancement of forward fluorescence

To measure the scattered light in the forward direction, we use the setup shown in the inset of Fig. 2a, to tightly focus and block the probe beam, while still collecting most of the atomic fluorescence on the CCD (charge-coupled device) camera. We focus the probe beam, after it interacts with the atoms, to a small spot with 15 μm root-mean-squared radius and block it using a beam stopping blade. We then translate the beam stopper perpendicular to the probe beam by a distance Δ*x* from our reference point of *x*=0, which we define as the position of the beam stopper where we see the greatest fluorescence without saturating the CCD camera with the probe beam. As only the forward direction is particularly sensitive to positional changes, we convert this change in position to a change in angle simply using 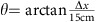, where the first lens with a 15-cm focal length collimates the fluorescence. In numerical calculations, the CCD camera is simulated as a ring area centred around the forward direction and the average intensity collected over the ring is determined. The external radius is set to be large enough to reach the angular region outside the interference cone and the inner angular radius *θ*_sim_, simulating the blocking of the signal by the beam stopper, is varied accordingly to the experiment. To account for the difference between *σ*_sim_ and the experiment cloud size, *θ*_sim_ is rescaled so that we satisfy the experimental observation that at *θ*_max_ the enhancement factor drops to 1.

## Additional information

**How to cite this article:** Bromley, S. L. *et al.* Collective atomic scattering and motional effects in a dense coherent medium. *Nat. Commun.* 7:11039 doi: 10.1038/ncomms11039 (2016).

## Supplementary Material

Supplementary Information Supplementary Figure 1, Supplementary Notes 1-2 and Supplementary References.
