# Supplementary material for: Collective atomic scattering and motional effects in a dense coherent medium
Source: Nat Commun. 2016 Mar 17;7:11039. doi: 10.1038/ncomms11039 (PMC4800430; doi:10.1038/ncomms11039)
Supplement: Supplementary Information — Supplementary Figure 1, Supplementary Notes 1-2 and Supplementary References. [file ncomms11039-s1.pdf]

Supplementary Figures

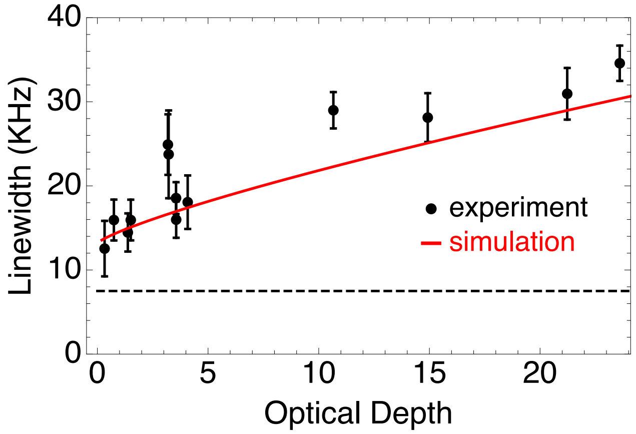

Supplementary Figure 1: Comparison between theoretical calculations and the linewidth data in Ref. [1]. The linewidth data were taken under the same condition as the red data in Fig. 4, where motional effects are significant (see Ref. [1] for experimental details). Still the theoretical model presented here can capture the linewidth broadening. Here $\eta = 1.5$ is used in the numerical simulations. All experimental errorbars are for statistical uncertainties.

Supplementary Note 1: On resonance optical depth

For the $J = 0 \rightarrow J = 1$ transition, the atom-photon scattering cross section is $\Sigma(\Delta) = \frac{6\pi}{k^2}\frac{1}{1+4(\Delta/\Gamma)^2}$, with k the wavevector of the photon and Δ, Γ are the detuning of the driving laser and the natural linewidth, respectively [2]. In the experiment, the atomic cloud has approximately a Gaussian distribution $n(x,y,z) = n_0 e^{-\frac{x^2}{2R_x^2}-\frac{y^2}{2R_y^2}-\frac{z^2}{2R_z^2}}$, where n_0 satisfies $\int dxdydzn(x,y,z) = N$, and N is the total number of atoms. Along the line of observation, *e.g.* $\hat{x}$, the on resonance optical depth is related to the resonant scattering cross section $\Sigma_0 = \frac{6\pi}{k^2}$, and the column density averaged

over the profile perpendicular to this direction [2,3],

$$\begin{aligned} OD &= [\int dydzn(y,z)]^{-1}\int dydzn(y,z)OD(y,z) & (1)\\ &= [\int dydzn(y,z)]^{-1}\int dydzn(y,z)\int dxn(x,y,z)\Sigma_0 & (2)\\ &= [\int dydzn(y,z)]^{-1}\int dydzn(y,z)e^{-\frac{y^2}{2R_y^2}-\frac{z^2}{2R_z^2}}\int dxn_0e^{-\frac{x^2}{2R_x^2}}\Sigma_0 & (3)\\ &= \frac{3N}{2k^2R_yR_z} & (4)\\ &= \frac{3N}{2k^2R_\perp^2}, & (5) \end{aligned}$$

which is the OD defined in the maintext.

Supplementary Note 2: Numerical simulation of the coherent dipole model

To simulate the experiment, we use $N_{\text{sim}} \sim 3000$ to 10000 atoms and assume they are randomly distributed according to a density distribution $n(x,y,z) \propto e^{-\frac{x^2}{2\sigma_x^2}-\frac{y^2}{2\sigma_y^2}-\frac{z^2}{2\sigma_z^2}}$, where $\sigma_{x,y,z}$ denote the widths of the atomic cloud in the simulation, and the aspect $\sigma_x : \sigma_y : \sigma_z$ is kept the same as the one measured in experiment. We average over a sufficient amount of configurations until convergence is achieved. To reproduce the behavior of the $N_{\text{exp}} \sim 10^7$ Sr atoms interrogated in the experiment, with the smaller number of atoms used in the numerical simulations we need to rescale the widths σ_α. For linewidth and fluorescence intensity, which are dominantly OD effects, the appropriate rescaling would be to match the OD used in the experiment and require $\sigma_\perp^{OD} = (N_{\text{sim}}/N_{\text{exp}})^{1/2}R_\perp$. However, under that procedure the density used in the theory does not match the experimental densities, instead it is much larger due to the factor $N_{\text{exp}}/N_{\text{sim}} \sim 10^4$, and this introduces non-negligible modification on the linewidth. We find that this issue can be compensated by a constant

rescaling of the width $\sigma_\perp^{\rm sim} = \eta\sigma_{OD}$. In the numerical simulation, we keep the parameter η constant to model all the experimental measurements taken under the same conditions. In Fig. 2(a) and (b), we set $\eta = 5$ for both the blue and red probe simulations under different detection angles θ and atom numbers. The experimental measurements shown in Fig. 2(c) and Fig. 3(a) (b) were taken under different geometries, and we use $\eta = 2.35$ for those simulations.

We account for motional effects in the red transition by introducing random detunings $\delta\nu$ for each atom and these are sampled according to a gaussian thermal distribution

$$P(\delta\nu) = \frac{1}{\sqrt{2\pi}\varDelta_{\rm D}}\exp\left(-\frac{\delta\nu^2}{2\varDelta_{\rm D}^2}\right). \tag{6}$$

Here $\varDelta_{\rm D}$ is the Doppler width at the experimental temperature. Specifically, for non-interacting two-level atoms, the atomic coherence is modified as

$$b_j = \frac{\Omega e^{i\mathbf{k}\cdot\mathbf{r}_j}}{(\varDelta - \delta\nu_j) + i\varGamma/2}. \tag{7}$$

For the incoherent scattering, this leads to

$$I_{\rm incoh} = \frac{1}{\sqrt{2\pi}\varDelta_{\rm D}}\int d\delta\nu_j |b_j|^2 e^{-\delta\nu_j^2/2\varDelta_{\rm D}^2}, \tag{8}$$

while for the coherent scattering in the forward direction, one has to take into account pairwise atomic contributions, such that

$$I_{\rm coh} = \frac{1}{2\pi\varDelta_{\rm D}^2}\int d\delta\nu_j d\delta\nu_{j'} b_j b_{j'}^* e^{-\delta\nu_j^2/2\varDelta_{\rm D}^2} e^{-\delta\nu_{j'}^2/2\varDelta_{\rm D}^2}, \tag{9}$$

thus the on-resonance enhancement factor is

$$\frac{I_{\rm coh}}{I_{\rm incoh}} = \frac{\sqrt{\frac{\pi}{2}} e^{\frac{1}{8\varDelta_{\rm D}^2/\varGamma^2}} \,{\rm Erfc}(\frac{1}{2\sqrt{2}\varDelta_{\rm D}/\varGamma})}{2\varDelta_{\rm D}/\varGamma}, \tag{10}$$

where Erfc is the complementary error function. This shows a suppression of the forward interference that depends on $\varDelta_{\mathrm{D}}/\varGamma$.

A similar procedure was used in Refs. [3, 4] to deal with motional effects. This simple treatment of the atomic motion accounts only for the Doppler shifts experienced by the atoms, but neglects light induced mechanical effects on atoms and random phase evolution in the dipole-dipole coupling due to atomic motion [3]. We expect it to be valid if the atoms are weakly driven, $\Omega \ll \varGamma$, and for low velocities $kv \leq \varGamma$ or short probing times $t \lesssim 1/kv$. Those conditions are more or less satisfied in the red measurements presented in Figs. 2 and 3. The random dephasing added in the coherent dipole model gives rise to a Voigt profile lineshape with a constant Gaussian width that is consistent with $\varDelta_{\mathrm{D}}$, and with a Lorentzian width that increases with OD. In Fig. 3(c), an $\eta = 2.35$ is used in the simulations for all the different OD conditions.

For treating the density shift, which is dominantly a density effect, it is more appropriate to rescale the cloud size to match the experimental density, $\bar{\sigma}^{\mathrm{density}} = (N_{\mathrm{sim}}/N_{\mathrm{exp}})^{1/3}\bar{R}$. Here $\bar{\sigma}$ and $\bar{R}$ are the corresponding geometric means. This is the procedure we use to produce the theory data presented in Fig. 4. In this case, nevertheless, the frozen dipole approximation is not able to capture the large density shift observed in the red probe experiment. The model is only able to reproduce the blue probe density shift. The red frequency shift measurements were performed under different conditions than those used for Figs. 2 and 3 (see Ref. [1]). The failure of the frozen dipole model to reproduce the density shift in situations when atoms are allowed to be a significant amount of time in the excited state and closely approach to each other in a

collision event, emphasizes the need to fully model the interplay between short and long ranged interactions, and atom motion in a dense sample [5]. While such effects are crucial for the frequency shift in atomic emission [4], in our calculations, the collective linewidth broadening turns out to be less affected. In Supplementary Fig. 1, the numerical results obtained with the same procedure as described above are compared with the red transition linewidth data measured together with the density shift (see Fig. 4) in Ref. [1]. The fair agreement between theory and experiment suggests that, even when motion is important, the frozen dipole model is capable of capturing some of the relevant features of collective atomic emission in a dense medium.

Supplementary References

1. Ido, T. *et al.* Precision spectroscopy and density-dependent frequency shifts in ultracold Sr. *Phys. Rev. Lett.* **94**, 153001 (2005).

2. Chomaz, L., Corman, L., Yefsah, T., Desbuquois, R. & Dalibard, J. Absorption imaging of a quasi-two-dimensional gas: a multiple scattering analysis. *New Journal of Physics* **14**, 055001 (2012).

3. Bienaimé, T., Piovella, N. & Kaiser, R. Controlled dicke subradiance from a large cloud of two-level systems. *Phys. Rev. Lett.* **108**, 123602 (2012).

4. Javanainen, J., Ruostekoski, J., Li, Y. & Yoo, S.-M. Shifts of a resonance line in a dense atomic sample. *Phys. Rev. Lett.* **112**, 113603 (2014).

5. Trippenbach, M., Gao, B., Cooper, J. & Burnett, K. Slow collisions between identical atoms in a laser field: The spectrum of redistributed light. *Phys. Rev. A* **45**, 6555–6569 (1992).
